# Lay Not Thy Hand to the Plowing Beast

**Published:** 1890-04

**Authors:** 


### "LAY NOT THY HAND TO THE PLOWING BEAST."

BY A VETERINARY SURGEON.

In the common interest of humanity, I feel compelled to offer a few remarks, regarding a most barbarous act of cruelty, perpetrated upon horses and mules, viz.: Burning, by means of a *red-hot iron*, the "bars" or "ridges" situated in the roof of the mouth, constituting the "hard palate," for the condition known as "Lampas."

Now, lampas is neither more nor less than a swollen condition of this part of the roof of the mouth, due to congestion and inflammation, during the period of teething, seen in almost all young "solipeds" at the time they shed their temporary, or colt teeth, to become possessed of their permanent, horse teeth. It occasionally occurs in older horses and mules, and is usually due to some error in feeding—it may be over-feeding.

Imagine for one moment a human medical practitioner taking a red-hot iron and with it burning the swollen and inflamed gums of a child under similar circumstances. And yet the conditions are almost identical. The only difference being, that one is a human being, the other a poor dumb brute.

It is sad to relate, but nevertheless true, that this cruel practice has been in operation for ages, and in nearly every country in the world; it is sincerely to be hoped that such painful and unneccessary torturing will be die a natural death, and that people will take a little more time to consider before subjecting these noble animals to such inhuman treatment, or rather maltreatment. Not only does it occasion excruciating agony to the dumb sufferer at the time, but the after consequences in many cases are of a most grave and serious nature, as "*tetanus*," commonly known as lock-jaw, frequently results.

All the treatment that is necessary in most cases is to give the animal a dose of purgative medicine, such as a ball composed of four or five drachms of Barbadoes aloes; wash the mouth with an astringent solution made by dissolving ¾ oz. alum and 1 oz. borax in a quart of water; or, it may be, carefully scarifying the congested part with a *clean*, sharp pointed lancet or pocket knife, to relieve that congestion; all the time allowing the animal to have soft, easily masticated food only.

Should the above remarks have the effect of preventing, at any rate to some extent, this horrible practice, and thereby saving such unnecessary and agonizing suffering to some of man's best friends, the writer will be amply repaid.

